# How decision makers can use quantitative approaches to guide outbreak responses

**DOI:** 10.1098/rstb.2018.0365

**Published:** 2019-05-20

**Authors:** Oliver Morgan

**Affiliations:** Department of Health Emergency Information and Risk Assessment, Health Emergencies Programme, World Health Organization, Geneva, Switzerland

**Keywords:** decision-making, outbreaks, infectious diseases, modelling

## Abstract

Decision makers are responsible for directing staffing, logistics, selecting public health interventions, communicating to professionals and the public, planning future response needs, and establishing strategic and tactical priorities along with their funding requirements. Decision makers need to rapidly synthesize data from different experts across multiple disciplines, bridge data gaps and translate epidemiological analysis into an operational set of decisions for disease control. Analytic approaches can be defined for specific response phases: investigation, scale-up and control. These approaches include: improved applications of quantitative methods to generate insightful epidemiological descriptions of outbreaks; robust investigations of causal agents and risk factors; tools to assess response needs; identifying and monitoring optimal interventions or combinations of interventions; and forecasting for response planning. Data science and quantitative approaches can improve decision-making in outbreak response. To realize these benefits, we need to develop a structured approach that will improve the quality and timeliness of data collected during outbreaks, establish analytic teams within the response structure and define a research agenda for data analytics in outbreak response.

This article is part of the theme issue ‘Modelling infectious disease outbreaks in humans, animals and plants: epidemic forecasting and control’. This theme issue is linked with the earlier issue ‘Modelling infectious disease outbreaks in humans, animals and plants: approaches and important themes’.

## 1. What is decision-making for outbreak responses?

The process of decision-making during outbreak responses is not well described. Decision makers are responsible for selecting the types and composition of public health interventions, directing the deployment of staff and logistical capacities, summarizing key messages to communicate to professionals and the public, forecasting future response needs, and establishing strategic and tactical priorities along with their funding requirements. However, making leadership decisions during an outbreak responses can sometimes feel like operating in a ‘data-free’ zone, especially at the start of an outbreak when there may be little epidemiological information available or when the causative agent is not yet known. Decision makers may also contend with an incomplete understanding of the cultural or political context where the event is occurring, uncertainty around the impact and relative advantage of different disease control measures, or delays in reports of operational information from on-the-ground responders.

In the absence of sufficiently complete and timely data, decision makers usually draw on experiential knowledge from previous outbreaks, partially from their own experience, but also through consulting disease experts who are also knowledgeable about the published and unpublished literature. While this is a pragmatic approach to bridging the data void, there are some drawbacks. There can be considerable variation in the recommendations from disease experts because they do not all have the same level of experience, have different interpretations of the published and unpublished data and may have differing abilities to apply data for decision-making in new outbreaks. A second limitation is that the ‘expert opinion’ approach is not explicit about what the key variables or factors are that need to be taken into account when making decisions, which in turn makes it hard for non-disease experts to engage in the decision-making process; decision makers must therefore have ‘faith’ that the disease experts are right. A third limitation is that experts often put a lot of emphasis on one scientific discipline, such as epidemiology or virology, usually occluding other important disciplines like anthropology, behaviour change, economics, etc. In the end, it is left to decision makers to rapidly synthesize data from different experts across multiple disciplines, bridge data gaps as best they can and translate epidemiological analysis into an operational set of decisions for disease control: this is what makes leadership in outbreak responses so challenging.

Over the last 10 or so years, there has been considerable progress in the use of analytic approaches to support decision makers in the control of infectious diseases [1]. There are many examples of decision support using statistical modelling from disease control programmes such as vaccination [2], TB control [3] and prevention of HIV [4]. There have also been many important examples of analytic approaches to help decision makers during outbreak responses. A particularly high-profile example of this was the forecasting of the West Africa Ebola outbreak by Meltzer *et al*. [5], which was a key factor in the decision of the US government to deploy its military to Liberia to support outbreak control activities. However, despite some notable successes, we continue to fail to apply analytic approaches systematically in outbreak responses. This is largely because decision makers themselves have not clearly described what support they need. In this article, I define what decision makers need and how analytic approaches can be systematically used to support them for better decision-making during outbreak responses.

## 2. Quantitative approaches needed for decision-making in outbreak responses

Quantitative approaches cover a large number of different aspects of working with data, including data management, visualization, statistical analysis, modelling, machine learning (ML) and geospatial analysis [6–8]. Applying quantitative approaches to decision-making for outbreak responses has the potential to overcome many of the limitations mentioned above by assisting decision makers to use available data optimally. Formulating decisions more explicitly also helps engage multiple stakeholders in the decision-making process and aids communication to interested parties and the public.

While decision-making for outbreak responses is a highly iterative process, it is helpful to characterize how analytic approaches could help in distinct phases of outbreak responses. Table 1 summarizes three phases of outbreak responses: investigation, scale-up and control. A fourth phase of enhanced surveillance once the outbreak is over could also be defined [9], but the tools are the same as for routine surveillance outside of a response and so I have not included it here. The *investigation phase* is the earliest part of a response and can be characterized as having the greatest uncertainty in terms of understanding the epidemiology of the outbreak. During this phase, case–patient data may be limited and collected retrospectively for patients who have died or recovered. Data visualizations are the most helpful for decision makers during this phase, giving an early indication of the extent of the outbreak. It may be possible to do some preliminary statistical modelling to understand the transmission dynamics and make limited forecasts with parameters from previous outbreaks. Hypotheses about the epidemiology that may need testing are often identified in this phase. The *scale-up phase* is inherently operational, with significant effort going into deployment of teams, logistics and establishing systems. Implementing robust data collection systems is the critical enabling step for analytics during the scale-up phase. The systems that get established in this phase, however imperfect, often endure to the end of the response. Input from data scientists can improve the data collection, and cleaning and data linkage systems, using tools such as R and Python to automate much of the data management work. More granular data collected in this phase can facilitate further statistical modelling and response planning. The *control phase* is the longest part of a response and is characterized by an increasingly sophisticated response. Strong monitoring and ongoing improvements in control interventions best characterize the control phase. Modelling can be particularly helpful in designing control strategies, as well as gaining yet further insights into disease transmission, such as through analysis of genetic sequences of pathogens. Testing hypotheses about risk factors is also most likely to be achievable in this phase, which in turn should inform the composition of interventions. Using data visualization tools for response monitoring dashboards is invaluable to decision makers so they can quantitatively track the quality of response operations and adjust the response accordingly.

**Table 1.** Response actions, analytic approaches to aid decision makers and examples of analytic tools.

	response actions	areas where analytic approaches could help decision makers	examples of analytic tools
investigation phase (days or weeks)	deployment of investigation teams verification and validation of initial information reported	data visualization of person, place and time quantifying transmission dynamics generation of hypotheses for further analysis	ArcGIS and R for visualization and geospatial analysis statistical modelling with aggregated event data and parameters from previous outbreaks
scale-up phase (weeks)	deployment of response team define strategic response logistical movement of material establishment of data management systems implement initial control measures	data systems design and linkage data cleaning and reporting routines forecasting size of the event planning the size and extent of response	online and handheld device data collection systems R and Python scripts for data cleaning and linkage forecasting using patient-level data scenario simulations for response planning
control phase (weeks or months)	sustained response interventions tailored to context rigorous monitoring of response performance testing hypotheses	further detailed epidemiological descriptions characterization of transmission dynamics investigation of hypotheses about risk factors designing intervention strategies estimating end of outbreak	ArcGIS for response monitoring dashboards statistical modelling of intervention strategies risk assessment for neighbouring unaffected areas phylogenetic tree analysis of virus relatedness

As can be seen from table 1, many quantitative approaches can be applied during more than one of the response phases. For the following discussion, I have therefore summarized the broad categories of quantitative approaches for outbreak response as: improved quantitative methods for epidemiological descriptions of outbreaks; investigations of causal agents and risk factors; tools to assess response needs; identifying and monitoring optimal interventions or combinations of interventions; and forecasting for response planning.

### (a) Epidemiological descriptions of outbreaks

At the heart of epidemiology is the description of disease by person, place and time [10]. An iconic example of this is the investigation of cholera in London conducted by John Snow in 1854, which is now considered to be the first modern epidemiological investigation [11]. Good descriptive epidemiology is extremely powerful for decision makers as it can quickly guide the direction of an outbreak response. In fact, in many instances, descriptive analysis alone is sufficient for guiding disease control efforts in the early phase of an outbreak and more sophisticated analysis sometimes only provides marginal gains for decision makers, often because the results are usually available too late to be operationally relevant.

Tools for visualization of public health data beyond the traditional person, place and time epidemiological descriptions are developing extremely quickly, especially within the R environment [12]. The presentations of Hans Rosling are a great example of how visualizations and animations of data can be very powerful ways of interrogating and communicating data [13]; in a recent example, for the response to an outbreak of Ebola virus in North Kivu, Democratic Republic of the Congo (DRC), we used R to create dynamic transmission chain diagrams that allowed decision makers to interact with the data in many different ways. Further development of R packages could improve how we look at not just cases, but also contacts and perhaps other risk factor data. For the Ebola outbreak, we also created animations that not only increased our understanding of how the outbreak was spreading along major road networks; additionally, we were able to include data about community resistance to show how resistance was concentrated in some communities or families, which drove further transmission. These visualizations helped decision makers adjust interventions to screen travellers along major road networks, prepare healthcare providers in towns and cities where cases might travel to recognize and report patients with suspected Ebola virus disease (EVD), and to pinpoint community engagement efforts.

Another aspect that can be assisted with better approaches and tools is the management of data from responses. Optimal decision-making depends on high-quality data available in a timely fashion: poor quality data may result in the wrong decisions and slow data availability means that data are unavailable during the operationally relevant time window. Unfortunately, the complexities of data management are often underestimated and sufficient numbers of people and specialists dedicated to data management are frequently absent during emergency responses. Data management for outbreak responses is increasingly complex as larger quantities of different types and formats are collected, often requiring linkage between many different data collection systems, such as surveillance systems, clinical data, laboratory diagnostics, contact follow-up and so on. Additionally, data may be collected in many different locations and using different data collection systems. Faster, more reliable data management can speed-up the generation of good descriptive summaries and other analyses, and by extension decisions. Data science tools such as R and Python can automate and reduce time for data cleaning, management and preparation, which often consume a considerable amount of time for epidemiologists. Imputation and matching algorithms can also be used for filling data gaps, which are common during outbreak investigations, as well as faster and more robust merging of datasets without unique IDs, which is still an unfortunately common occurrence. During the 2014–2016 West Africa Ebola outbreak, considerable data cleaning was needed before the estimation of transmission parameters by Imperial College, the WHO Collaborating Center for infectious disease modelling [14]. The Imperial College team was able to set up robust data cleaning routines for data management and the ongoing analytic work throughout the outbreak. However, despite this example, decision makers rarely invest sufficient resources for robust data management on the ground during emergency responses, which consequently limits further analytic work by others.

### (b) Investigations of causal agents and risk factors

Epidemiological approaches to investigating the link between outbreaks and causative agents or exposures have traditionally relied on estimates of effect determined from study designs such as cohort studies, case–control studies and derivatives of these designs such as case–case studies, quasi-experimental methods [15] and spatio-temporal analyses [16]. While these analytic designs remain foundational for epidemiologists, advances in data science and analytic approaches enable other data sources to be incorporated in our epidemiological assessment of disease outbreaks and other health emergencies. For example, the growing availability of whole-genome sequencing (WGS) data, aided by the development of technology that enables WGS in the field, means that the relatedness of pathogen variants is increasingly being used to understand transmission dynamics in concert with other epidemiological information [17]. This can be particularly advantageous when self-report data from patients about exposures and contacts are not available, incomplete or inaccurate [18]. Working with WGS data is computationally intensive, requiring the processing of large volumes of data and the application of sophisticated data processing and analytic methods, which extend beyond the capacities of most epidemiologists and require input from data science.

ML is another potential tool for outbreak responses, although practical uses for outbreak analysis are still in their infancy. ML has been used to analyse prognostic risk factors for EVD outcomes, with the ability to account for missing data, which could be helpful for decision makers who wish to consider interventions that limit the impact of outbreaks [19]. ML has also been used as an efficient way to estimate disease transmission dynamics, which would be especially helpful when managing an outbreak of a new pathogen or variant [20]. The World Health Organization (WHO) currently employs ML for detecting signals about new public health events from vast amounts of online data, using the Epidemic Intelligence from Open Sources (EIOS) platform, applying natural language processing to process, categorize and assemble data. It is possible that these applications could be extended to analyse data from social media, purchasing patterns, travel data and qualitative data from other sources to gain greater insights into behavioural risk factors for outbreak control. In the future, ML could also be helpful for analysing large datasets from large or complex non-health sector data sources that may help us understand transmission risk factors, such as mobile phone data for movement patterns or remote sensing data for environmental exposures [21,22]. Combining these data sources with the analysis of other epidemiological data can increase our understanding of outbreaks.

### (c) Response needs

Effective outbreak responses require good logistical planning to ensure that the correct materials are available at the right time and in the right locations. Critical logistical requirements include medicines, vaccines, laboratory reagents, staff, physical infrastructure such as acute care beds, personal protective equipment, accommodation for staff, vehicles and so on. In addition to the quantity of supplies needed, the location about where to deploy supplies may also be critical. Underestimate the needs and people may die and the outbreak may be poorly controlled. Overestimating response needs may increase costs and resources that are deployed, potentially depriving other outbreak responses of vital supplies. Take too long to determine the needs and supplies may arrive late, retarding disease control measures and resulting in increasing needs beyond initial assessments. Clearly, avoiding these shortcomings in logistical planning is especially difficult when there is uncertainty about the magnitude and evolution of an outbreak [23]. By improving the accuracy and timeliness of quantitative estimates of an outbreak, the provision of supplies and medical services can optimize outbreak responses.

Decision makers can understand response needs through effective cooperation between disease modellers, operational planners and on-the-ground response teams. During the response to a large diphtheria outbreak in 2017 among the Rohingya displaced population in Cox's Bazar, Bangladesh, Médecins Sans Frontières, the London School of Hygiene and Tropical Medicine and WHO used epidemiological data collected by field teams to model the size of the outbreak and to estimate the necessary number of acute care beds and medical teams required to control it [24]. Quantitative approaches were also used during the West Africa Ebola outbreak to estimate isolation bed capacity [25]. A clear process for estimating response needs is also important for communicating budget requirements to donors as well as prioritizing allocation of resources such as vaccines when supply is limited.

### (d) Optimizing interventions

There are many different interventions used for managing outbreaks. These include vaccination, isolation of infectious patients, use of medicines such as antibiotics, airport screening, improving water supply, laboratory diagnostics, community mobilization and so on. Optimizing interventions is not only important for reducing disease transmission as quickly as possible, but also to avoid unnecessary use of scarce resources such as medical supplies, funds, or deployment of skilled personnel. It is also important to consider optimal locations for interventions to be deployed, such as laboratories or specialist treatment facilities, or the timing or sequence of interventions [11].

Modelling approaches have been used to optimize vaccine policy for disease prevention programmes, such as the introduction of human papillomavirus (HPV) vaccine [2]. Similar approaches have been applied for reactive vaccination interventions for outbreak control. For example, during 2018 in Yemen, the planning for oral cholera vaccination used modelling approaches in combination with epidemiological data from the outbreak in 2017 to select the optimal combination of districts to control the outbreak. During the Ebola outbreak in West Africa, modelling was used to consider how newly developed rapid diagnostic assays could be used to improve disease control; this is likely to be an especially helpful application of quantitative methods considering the increasingly rapid pace of technological development for diagnostics, vaccines and therapeutics [23]. During the 2018–2019 Ebola outbreak in North Kivu, DRC, modelling was used to consider how, under different security scenarios, outbreak control interventions could be optimally combined (WHO 2018, unpublished data). From an operational perspective, the application of quantitative methods to optimize interventions needs to incorporate contextual information into quantitative analyses, so that constraints, such as security limitations, local political considerations or access restrictions for logistical planning such as damaged roads and bridges, are taken into account. This can be done with analysts working closely with decision makers and other responders to iteratively refine analyses, which is probably most effectively achieved by having one of the analytics team deployed to the response operations on the ground.

### (e) Forecasting

Understanding the evolution of an outbreak is important for logistical planning, optimizing interventions and evaluating the effectiveness of existing interventions. There has been considerable investment of energy in developing approaches for infectious disease forecasting [26]. However, there is still limited evidence of how such forecasts contribute to improved decisions for outbreak management. Part of the challenge is that despite the advances in outbreak forecasting, the uncertainty in the forecasts continues to be too big to use as a basis for operational decisions, especially early on in an outbreak [27]. Another challenge is the disconnect between those producing forecasts and decision makers; the link between the two professional communities is often limited or non-existent. This leads to modellers making forecasts that decision makers may not be aware of or do not understand. Establishing better connections between these professional communities requires ongoing collaboration and joint training. RECON (the R Epidemics Consortium) is an excellent example of an initiative that brings modellers, statistical programmers and emergencies responders together. An example of this type of collaboration is published in this journal special issue and shows how modelling the end tail of an Ebola outbreak can be used for public health decision-making [9].

Yet, another challenge for decision makers is that there is a proliferation of modelling groups producing forecasts, sometimes using aggregated data published in situational reports performed independently from the responding organizations. Decision makers can find it difficult to know how to handle multiple forecasts, especially as any one of the approaches is not necessarily superior. An evaluation framework to assess multiple forecasts and their robustness, such as including validation or sensitivity analysis, may be helpful for decisions makers [28]. Different forecasts may also complicate decision-making when divergent forecasts are used by different responding agencies or published in the media. Finally, even when forecasting models are well constructed, they may not address operationally relevant issues for decision makers. For example, there may be insufficient data for forecasts to be produced at operationally relevant geographical levels; in addition to knowing if an outbreak is going to get bigger, decision makers also need to know where the increase will occur. Understanding what data are needed by modellers may help on-the-ground response teams adjust their data collection approaches accordingly.

## 3. Future opportunities and challenges for improved decision-making during outbreaks

We need a greater understanding of what decisions are made and how they are taken during outbreak responses. Data about decision-making could be captured prospectively as a response progresses or we could use an after-action review to evaluate the decision-making process. Retrospective reviews of previous outbreaks conducted with the relevant decision makers could also generate additional insights. With a better knowledge about decision-making, we could further develop a framework for decision-making along the lines of the one presented in this paper (table 1). Using our framework, we can simultaneously develop our understanding of what analytic approaches could help decision makers and what data need to be collected by the field teams [29].

Writing a data collection plan might be a useful approach so that field teams and others providing remote support to the response can work in concert to ensure that decision makers and analysts have the most useful data in a timely way. In addition, having a data collection plan might help with properly resourcing data management in the field, which will address some issues of timeliness and quality of data. An analytics team should also be convened and at least one member should be assigned to work with key decision makers to help understand their needs and to facilitate communication of the outputs of the analytics team. Also, another member of the analytics team should be deployed to the field to work with decision makers on the ground and to understand important contextual factors. An analytics team should be recognized as a distinct entity from the epidemiology team because epidemiologists are involved in field activities such as case finding, surveillance, training and so on, and rarely have dedicated time to focus on analytic tasks. Over the medium term, a research agenda is needed, based on our decision-making framework, to focus partnerships with academic and other groups on the development of new decision-making approaches. In particular, further work on incorporating social science approaches for behavioural risk factors should be prioritized [30].

Here, I describe how data science and quantitative approaches can improve decision-making for responses to outbreaks of diseases that affect people. However, the approaches that I discuss can equally be applied to outbreaks of animal and plant diseases. Nonetheless, to realize the benefits of quantitative methods for decision-making, an explicit approach is needed within the incident management system to drive improvement in the quality and timeliness of data collection, to ensure that there is a dedicated analytic team, and to promote understanding and use of analytic outputs by decision makers.

## Data accessibility

This article has no additional data.
